# Human fetal skin derived merkel cells display distinctive characteristics *in vitro* and in bio-engineered skin substitutes *in vivo*

**DOI:** 10.3389/fbioe.2022.983870

**Published:** 2022-09-15

**Authors:** Katarzyna Michalak-Micka, Dominic Rütsche, Luca Mazzone, Vanessa L. Büchler, Ueli Moehrlen, Agnes S. Klar, Thomas Biedermann

**Affiliations:** ^1^ Tissue Biology Research Unit, Department of Surgery, University Children’s Hospital Zurich, Zurich, Switzerland; ^2^ Children’s Research Center (CRC), University Children’s Hospital Zurich, Zurich, Switzerland; ^3^ Spina Bifida Center, University Children’s Hospital Zurich, Zurich, Switzerland; ^4^ The Zurich Center for Fetal Diagnosis and Therapy, University of Zurich, Zurich, Switzerland; ^5^ Faculty of Medicine, University of Zurich, Zurich, Switzerland

**Keywords:** human fetal skin, Merkel cell, CK20, skin substitute, skin tissue engineering

## Abstract

Human skin contains specialized neuroendocrine Merkel cells responsible for fine touch sensation. In the present study, we performed in-depth analysis of Merkel cells in human fetal back skin. We revealed that these Merkel cells expressed cytokeratin 20 (CK20), were positive for the neuroendocrine markers synaptophysin and chromogranin A, and the mechanosensitive ion channel Piezo2. Further, we demonstrated that Merkel cells were present in freshly isolated human fetal epidermal cells *in vitro*, and in tissue-engineered human dermo-epidermal skin substitutes 4 weeks after transplantation on immune-compromised rats. Merkel cells retained the expression of CK20, synaptophysin, chromogranin A, and Piezo2 after isolation and in culture, and in the skin substitutes after transplantation. Interestingly, we observed that in fetal skin and in skin substitutes, only Merkel cells were positive for CK8, while in culture, also non-Merkel cells showed positivity for CK8. In summary, human fetal Merkel cells showed phenotypical features confirming their cell identity. This findings are of pivotal importance for the future application of fetal tissue-engineered skin in clinics.

## Introduction

Human Merkel cells were identified in 1875 by Friedrich S. Merkel. He attributed them a sensory function and therefore called them “Tastzellen” (touch cells) (Merkel, 1875). Today, these cells, known as Merkel cells, have been proven to display a mechanosensory function for gentle touch sensation (Maricich et al., 2009; Maksimovic et al., 2014). For this, Merkel cells form complexes with Aβ afferent nerve endings to transmit tactile stimuli (Narisawa and Hashimoto, 1991; Johnson, 2001; Ikeda et al., 2014). It was only recently revealed that these Merkel cell-neurite complexes are activated as a consequence of mechanotransduction via Piezo2 ion channels present on Merkel cells (García-Mesa et al., 2017; García-Piqueras et al., 2019).

The developmental origin of Merkel cells has been extensively debated in the past years. Nowadays, it is accepted that Merkel cells are of epithelial origin and localized in the stratum basale in the skin of most vertebrates (Morrison et al., 2009; Van Keymeulen et al., 2009). Interestingly, besides the epithelial origin of Merkel cells, these cells exhibit also typical neuroendocrine characteristics including the expression of chromogranin A and synaptophysin, and the presence of dense core granules (Iggo and Muir, 1969; Ortonne et al., 1988; Hartschuh et al., 1989).

Importantly, the expression of intermediate filaments, namely cytokeratins, differ in Merkel cells from keratinocytes. CK20 has been described to be an exclusive and unique marker for Merkel cells (Moll et al., 1993; Moll et al., 1995). Merkel cells exhibit also the expression of CK8 and CK18, however these two markers are also found in epidermal keratinocytes (Moll et al., 1984; Moll and Moll, 1992).

In adults, the highest numbers of Merkel cells was revealed in palmar skin, especially in the fingertips, i.e. regions highly involved in tactile sensation (Lacour et al., 1991). Interestingly, Merkel cells are already present in fetal human skin. They have been found especially in eccrine glandular ridges of fetal glabrous skin and in the basal layer of the developing infundibulum of hair follicles in fetal scalp skin (Moll et al., 2005). Further studies in human fetal skin revealed that Merkel cells represent a heterogeneous cell population (Xiao et al., 2014). In this respect, oval non-dendritic and non-oval dendritic Merkel cells were discriminated (Moll and Moll, 1992). The oval non-dendritic Merkel cells have been shown to be innervated and in contact to afferent nerve endings, whereas this was not the case for non-oval dendritic Merkel cells (Tachibana et al., 1997).

Gracia-Mesa et al. have shown that in the human fetal skin Merkel cells exhibit the expression of Synaptophysin, a typical presynaptic vesicle protein (García-Mesa et al., 2022b). In addition, the authors revealed the positivity of fetal Merkel cells for Piezo2, which is an essential component in mechanotransductive mechanisms. The fact that Merkel cells in fetal skin express Piezo2 and Synaptophysin as well as their innervation strongly suggests the ability of these cells to transduce tactile stimuli already during fetal development. However, additional functional assays are needed in order to confirm this hypothesis (García-Mesa et al., 2022a).

Despite the presence of Merkel cells in fetal skin, their isolation and culture have so far only be done using human adult skin biopsies. Merkel cells were still detected after passaging of epithelial cells in culture (Fradette et al., 2003; Hahn et al., 2019). Further, a study by Hahn et al. reported that Merkel cells were found in human skin substitutes applied in an animal model, suggesting that sensation could be restored using tissue-engineered grafts (Hahn et al., 2019).

In general, bio-engineered autologous dermo-epidermal human skin substitutes have been developed to treat large wounds such as burns, and have been successfully applied clinically by us and others (Boyce et al., 2017; Germain et al., 2018; Meuli et al., 2019). On the other hand, skin defects may also be present in congenital anomalies such as open spina bifida (myelomeningocele and myeloschisis), which is a devastating congenital malformation with an incidence of 1:1,000 worldwide (Copp et al., 2015; Möhrlen et al., 2020). In the last decade, fetal repair of open spina bifida has shown to yield better outcome results then postnatal repair and has therefore become as a standard of care in specialized centers (Moehrlen, 2021). However, in about 20%–30% of all cases a primary skin closure is not possible. The application of bio-engineered autologous skin grafts for skin closure during fetal repair could avoid complicated skin mobilization, flap construction, or even the use of temporary acellular skin substitutes (Mangels et al., 2000; Meuli et al., 2018; Mazzone et al., 2020). Including Merkel cells in the bio-engineered autologous skin grafts would potentially contribute to restoration of fine touch sensation after transplantation.

In this study, we therefore addressed the question if we can successfully isolate and culture Merkel cells from human fetal back skin while maintaining their neuroendocrine characteristics. Further, we examined if these cultured human fetal neuroendocrine Merkel cells are present and form complexes with afferent nerve endings in human tissue engineered skin substitutes after transplantation in our well-established animal model (Böttcher-Haberzeth et al., 2013; Mazzone et al., 2014; Biedermann et al., 2015; Klar et al., 2017; Michalak-Micka et al., 2019).

## Materials and methods

### Human fetal skin samples

Patient informed consent was obtained for the skin sampling as well as further investigations and experimental use. The study was authorized by the ethic committee of the Canton Zurich (KEK-ZH-Nr. 2015–0247 and BASEC No. PB_2020–00066) and conducted according to the Declaration of Helsinki Principles. Fetal skin biopsies were sampled during spina bifida aperta fetal surgery procedures at gestational week 24–25 for cell isolation and embedment in OCT compound (Sakura Finetek, Switzerland) with subsequent storage at - 20°C for later histological examination.

### Isolation and culturing of primary cells

Skin cell isolation and the following cultivation was performed as previously described in Böttcher-Haberzeth et al. (Böttcher-Haberzeth et al., 2013). Briefly, skin samples were reduced to small pieces (about 3 mm^3^) and digested in 12 U/mL dispase (BD Biosciences, Switzerland) combined with Hank’s balanced salt solution containing 5 mg/ml gentamicin (all from Invitrogen, Switzerland) at 4°C overnight. Thereafter, forceps where used to separate the epidermis and dermis for subsequent cell isolation. Epidermal cells were extracted from the epidermis by further digestion in 1% trypsin and 5 mM EDTA (both from Invitrogen, Switzerland) for 10 min at 37°C and afterwards cultured in serum-free keratinocyte medium containing 25 mg/ml bovine pituitary extract, 0.2 ng/ml epidermal growth factor, and 5 mg/ml gentamicin (all from Invitrogen, Switzerland). Further digestion of the dermal tissue in 2 mg/ml collagenase blend F (Sigma, Switzerland) for 4 h at 37°C maintained to the extraction of fibroblasts, which were further cultivated in Dulbecco’s modified Eagle’s medium (DMEM) containing 10% fetal calf serum (FCS), 4 mM L-alanyl-l- glutamine, 1 mM sodium pyruvate, and 5 mg/ml gentamicin (all from Invitrogen, Switzerland).

### Preparation of dermo-epidermal skin substitutes

Preparation of the dermo-epidermal skin analogues in six-well cell culture inserts with 3.0 μm pore size membranes, in a transwell system (BD Falcon, Switzerland), was performed as described in Biedermann et al. (Biedermann et al., 2015). In brief, the corresponding dermal compartment consisting of a membrane covered with collagen type I hydrogel containing human fetal fibroblasts was prepared first. Therefore, collagen type I (Symatese, France) was mixed with neutralization buffer containing NaOH and 1 × 10^5^ fibroblasts (passage 2) for polymerization. Those dermal analogues were further cultivated for 7 days in DMEM containing 10% FCS. After cultivation, 5 × 10^5^ keratinocytes (passage 2) were distributed onto each dermal equivalent. After one additional week of cultivation in SFM (Invitrogen, Switzerland), with medium change every second day, the dermo-epidermal skin substitutes were ready for transplantation.

### Transplantation of cultured dermo-epidermal skin analogs

The approval for the surgical procedure, performed on immuno-incompetent female nu/nu rats, 8–10 weeks old (Harlan Laboratories, Netherlands), was obtained from the local committee for Experimental Animal Research (permission number ZH 182/2016). The operation and transplantation procedure were conducted as described in Mazzone *et al.* (Mazzone et al., 2014). In brief, a full-thickness skin defect was prepared for the placement of the dermo-epidermal skin grafts. Additionally, to prevent wound closure from the surrounding rat tissue and therefore to protect the skin analogues, a surgical steel ring (diameter 2.6 cm) was sutured into the skin defect, using non-absorbable polyester sutures (Ethibond^®^, Ethicon, United States). Coverage with a silicone foil (Silon-SES, BMS, United States ) was used for further protection of the transplants, and gauze (Sincohaft, Puras, Switzerland) and tape (Leukoplast, BSN Medical, Germany) as standard wound dressing. Photographic documentation and dressing changes were conducted weekly. After 4 weeks *in vivo*, the skin analogues were excised and embedded in OCT compound for further analysis.

### Immunohistochemical staining and analysis of OCT embedded samples

Cryosections of OCT embedded fetal skin and skin analogue explants as well as freshly isolated and cultured fetal keratinocytes were further analysed by immunofluorescence staining (Pontiggia et al., 2009). Therefore, freshly isolated or cultivated keratinocytes (passages 0–2) were centrifuged to glass microscope slides, using attached cytofunnels (Shandon CytoSpin 4, Thermo Fisher Scientific, Switzerland). Per slide 200.000 cells were seeded, air dried, fixed in acetone/methanol 1:1 for 5 min at −20°C and stored at 4°C prior to the staining. Sliced cryosections (12 μm) were fixed and permeabilised using acetone/methanol 1:1 for 5 min at −20°C, subsequently air-dried, washed in phosphate-buffered saline (PBS, Invitrogen, Switzerland) and finally blocked in PBS containing 2% BSA (Sigma, Switzerland) for 30 min. Following primary antibodies were used for immunofluorescence analysis: CK20 (1:50, clone K20.8, Dako, Switzerland), Laminin 5 (Lam5, 1:50, clone P3H9-2, Santa Cruz, United States ), NF200 (200 kDa neurofilament, clone NF01, 1:50, Abcam, Germany), Ki67 (1:50, clone B56, BD Pharmingen, Switzerland), CK8 (1:50, K8.8, Abcam, Switzerland), Chromogranin A (1:50, clone C-12, Santa Cruz, United States ), Synaptophysin (1:50, clone SY38, Progen, Germany), CK15 (1:50, clone LHK15, Chemicon, Switzerland), CK10 (1:50, clone DE-K10, Dako, Switzerland), Piezo2 (1:50, NBP1-78624, Novus Biologicals, Switzerland).

After incubation with primary antibodies for 45 min in 2% BSA in PBS, slides were washed 3 times for 5 min. Thereafter the staining was continued by blocking with 2% BSA in PBS for 15 min and incubation with secondary antibody for 1 h in 2% BSA in PBS, followed by 5 min washing of the slides for three times and additional blocking with 2% BSA in PBS for another 15 min before incubation with additional primary antibody.

Secondary TRITC- or FITC- (Abcam, Germany) conjugated polyclonal goat *F* (abʹ)2 antibodies were used to visualize the primary antibody or Alexa 555-conjugated polyclonal goat *F* (abʹ)2 fragments were used to pre-label the primary antibodies according to the manufacturer’s instructions (Zenon Mouse IgG Labelling Kit, Molecular Probes/Invitrogen, Switzerland). For additional visualization of the cell nuclei, all slides were incubated for 5 min in PBS containing 1 μg/ml Hoechst 33342 (Sigma, Switzerland), washed twice for 5 min in PBS, and finally mounted with Dako mounting solution (Dako, Switzerland).

### Fluorescence microscopy

Microscopic evaluation of the immunohistochemical staining was performed using a DXM1200F digital camera connected to a Nikon Eclipse TE2000-U inverted microscope equipped with filter sets for Hoechst 33342-, FITC-, and TRITC (Nikon AG, Switzerland; Software: Nikon ACT-1 vers. 2.70). The elaborated graphical material was further processed with Photoshop 11.0 (Adobe Systems Inc., Germany).

### Relative quantification of CK20 expressing cells

The fraction of Merkel cells in primary freshly isolated keratinocytes (freshly isolated) and during *in vitro* culturing at the point of passaging (0P and P1) was determined by analyzing 100′000 cells using a Shandon CytoSpin 4 (Thermo Fisher Scientific, Switzerland). To control for donor variability, several primary cultures were analyzed (n = 6). After depositing cells on coated DoubleCytoslide slides (Thermo Fisher Scientific, Switzerland), cells were fixed for 5 min in ice-cold acetone/methanol and stained for CK20. Fluoroshield with DAPI (Sigma, Switzerland) was used to mount coverslips and counterstain nuclei. CK20 positive cells were manually identified and counted using a Nikon Eclipse TE-2000-U confocal microscope (Nikon AG, Switzerland; Software: Nikon ACT-1 vers. 2.70). Statistical analysis was used to compare CK20 positive fractions. Ordinary one-way ANOVA with Tukey’s multiple comparison test was used to analyze differences in population means (Prism 8, Vers. 8.3.1). A *p*-value <0.05 was considered statistically significant.

### Quantification of Merkel cells in fetal skin and dermo-epidermal skin substitutes

Merkel cells in human fetal skin and in tissue-engineered skin sections were identified and counted manually based on CK20 expression using ImageJ software. The data are presented as the number of CK20^+^ Merkel cells per 1 mm of basal cell layer. In each case counts were performed using five microscopic fields (×40 magnification) from two independent biological donors. Data are presented as mean ± SD.

### Quantification of oval and dendritic Merkel cells in fetal skin

Merkel cells in human fetal skin sections were identified and counted manually based on CK20 expression using ImageJ software. Subsequently, oval and dendritic Merkel cells were quantified over total number of CK20^+^ Merkel cells. Five microscopic fields at × 40 magnification were used in each group (*n* = 5 per condition; from three independent donors). Data are presented as a mean ± SD.

## Results

### CK20 expressing Merkel cells in human fetal skin

**FIGURE 1:**
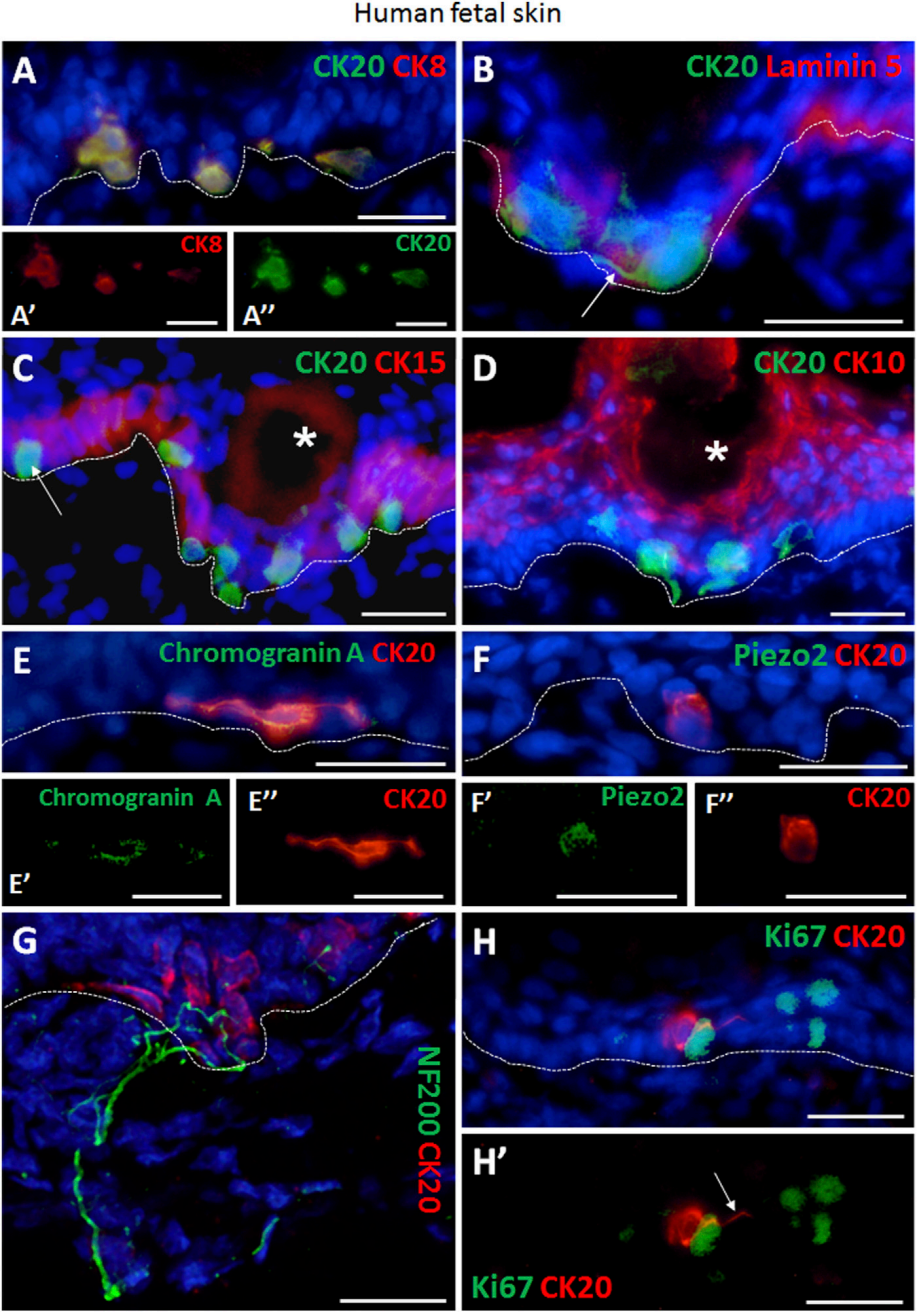
Immunofluorescence staining of human fetal back skin. **(A,A′,A″)** CK20 and CK8 double positive Merkel cells (**A**, CK20 green, CK8 red). **A′** and **A″** display single staining of CK8 (red) or CK20 (green) of Merkel cells shown in **(A)**. **(B)** CK20-positive (green) Merkel cells and basement membrane component Laminin 5 (red). White arrow depicts dendritic protrusion of a non-oval Merkel cell. **(C)** CK20-positive (green) Merkel cells and CK15-positive (red) basal layer keratinocytes. White arrow indicate a non-dendritic oval Merkel cell in interfollicular epidermis. White asterisk marks a hair follicle. **(D)** Merkel cells (CK20, green) and CK10-positive (red) suprabasal layer keratinocytes. White asterisk denotes a hair follicle. **(E,E′,E″)** Expression of neuroendocrine marker chromogranin A (green) in CK20-positive (red) Merkel cell. **E′** and **E″** display single staining of chromogranin A (green) or CK20 (red) of Merkel cell shown in **(E). (F,F′,F″)** Presence of Piezo-type mechanosensitive ion channel component 2 (Piezo2, green) in CK20-positive (red) Merkel cell. **F′** and **F″** depict single staining of Piezo2 (green) or CK20 (red) of Merkel cell shown in **(F)**. **(G)** Neurofilament 200 (NF200, green) nerve endings projecting to Merkel cells (CK20, red). **(H,H′)** Ki67 (green) as marker for proliferating cells. CK20-positive (red) Merkel cells are Ki67-negative. **(H′)** displays Ki67 (green) and CK20 (red) double staining without cell nuclei Hoechst dye. White arrow denotes dendritic protrusion of Merkel cell. Dashed white lines indicate locations of dermal-epidermal junctions. Hoechst 33342 (blue) was used to counterstain nuclei. Scale bars: 25 µm.

We performed combined immunofluorescence co-staining against cytokeratin 8 (CK8) and CK20 in order to visualize Merkel cells in skin tissue sections from 24 to 25-week old human fetuses (Figure 1A–A’). In addition, the epidermal basal layer localization of CK20 positive Merkel cells was further confirmed by an immunofluorescence co-staining with Laminin 5, a basement membrane marker (Figure 1B). In fetal skin, migrating Merkel cells still exhibited dendritic protrusions (white arrow, Figure 1B), whereas a more oval shaped cells were visible only when they reached maturation at their target location in the basal cell layer (white arrow, Figure 1C). Quantification revealed that the number of oval and dendritic Merkel cells in human fetal skin is similar (50.9 ± 6.8% vs. 49.1 ± 6.8%, respectively, *p* > 0.05, not significant) (Supplementary Figure S1A).

Furthermore, CK20-expressing Merkel cells were found in close proximity to CK15 positive basal layer keratinocytes (Figure 1C). The different localization of basal Merkel cells from the suprabasal terminally differentiated keratinocytes was verified by CK20 and CK10 co-staining (Figure 1D).

Moreover, we confirmed the neuroendocrine nature of Merkel cells by the expression of Chromogranin A in all CK20^+^ cells (Figures 1E–E’’). Further, the presence of the mechanosensitive and stretch-gated ion channel Piezo 2 (Piezo-type mechanosensitive ion channel component 2) was also detected in CK20^+^ Merkel cells (Figures 1F–F’’). Interestingly, distinct shapes of Merkel cells were observed in the skin sections of human fetuses, particularly oval and dendritic cells. The innervation of matured oval-shaped CK20^+^ Merkel cells was further visualized by presence of neurofilament 200 (NF200) positive nerve endings (Figure 1G; Supplementary Figures S3A–C), as NF200 is a late-stage marker for mature afferents. In contrast, non-matured Merkel cells were not associated with NF200^+^ peripheral nerve endings (Supplementary Figures S2A–C) and exhibited dendritic phenotype.

Importantly, we did not detect Ki67 expression in CK20^+^ Merkel cells (Figures 1H–H’), demonstrating that Merkel cells were not in a cell cycle.

### CK20 expression is maintained in cultured human fetal skin derived Merkel cells *in vitro*

**FIGURE 2:**
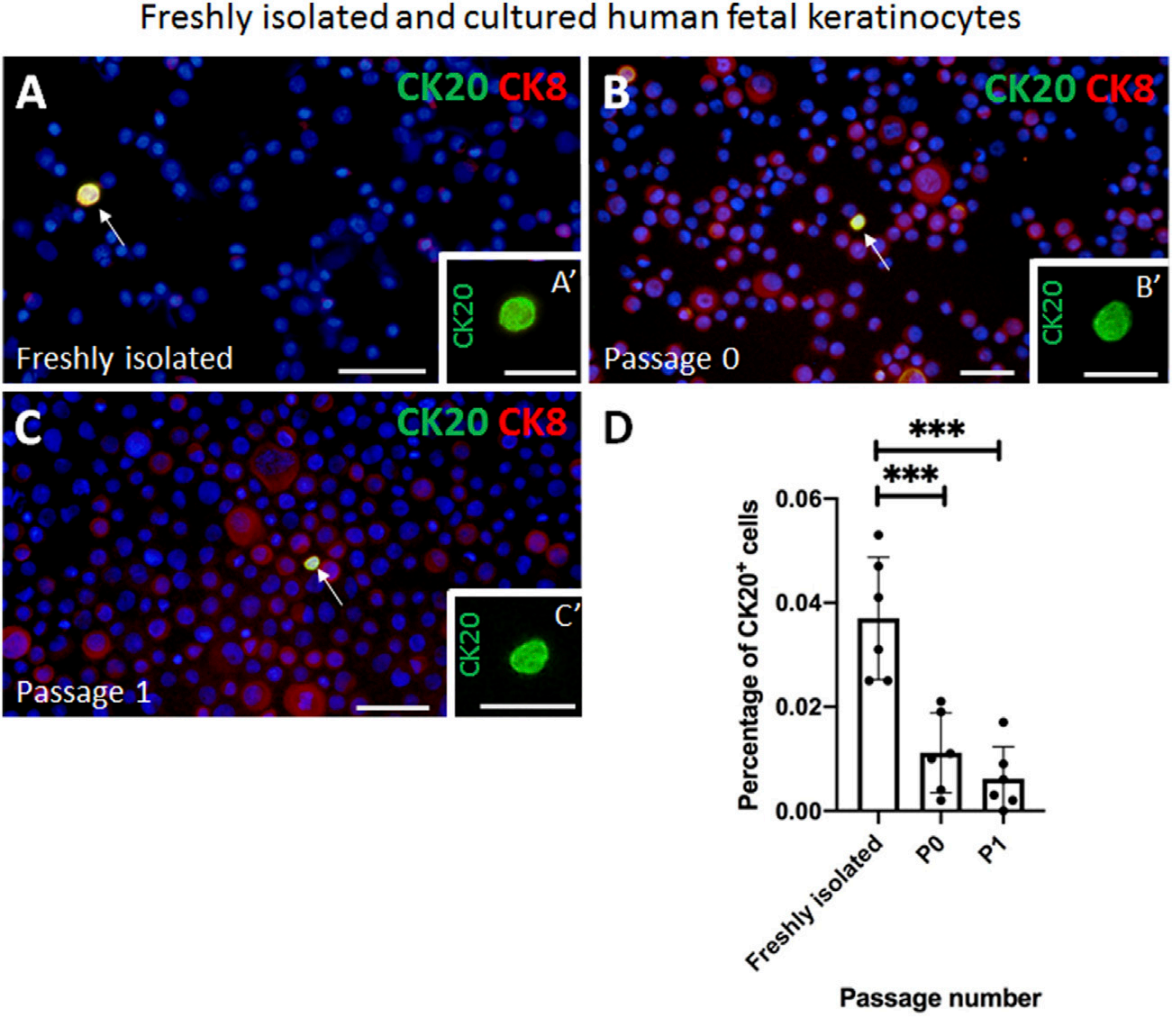
Examples of immunofluorescence staining of freshly isolated and cultured cytospinned keratinocytes from human fetal skin. **(A,A′)** CK20 (green) and CK8 (red) double positive Merkel cell is present in freshly isolated keratinocytes. **(A′)** shows a magnification of the CK20-positive (green) Merkel cell shown in **(A)**. **(B,B′)** CK20-positive (green) Merkel cell is present in cultured cells (Passage 0). Note the expression pattern of CK8 (red) in non-Merkel cells. **B′** indicates the CK20-positive (green) Merkel cell displayed in **(B)**. **(C,C′)** Expression of CK20 (green) in cultured cells (passage 1 in **C**, **C′**). CK8 (red) is also expressed in non CK20-positive Merkel cells. **(C′)** displays the CK20-positive (green) Merkel cell in **(C)**. **(D)** The fraction of CK20-positive Merkel cells decreases during *in vitro* culturing. Statistical analysis shows a steady decrease of CK20-positive Merkel cells from 0.037% ± 0.012% freshly isolated cells to 0.011% ± 0.007% (Passage 0, P0) and to 0.006% ± 0.006% (Passage 1, P1). The decrease is most significant between freshly isolated cells and Passage 0 (*p* < 0.001) and between freshly isolated cells and Passage 1 (*p* < 0.001). Ordinary one-way ANOVA with Tukey’s multiple comparison test, *n* = 6 per group, ***: *p* < 0.001. Hoechst 33342 (blue) was used to counterstain nuclei. Scale bars: A, B, C, D = 50 μm, A′, B′, C′, D’ = 25 µm.

Next, we isolated epithelial cells from human fetal back skin biopsies and we cultured them on tissue culture plastic. We investigated the percentage of CK20-expressing cells directly after isolation and in the cultured keratinocytes at passage 0 (P0) and 1 (P1) (Figures 2A–D).

Immunofluorescence staining of cytospinned keratinocytes directly after isolation revealed that 0.037 ± 0.012% of all cells were positive for CK20. In culture, the percentage of CK20-expressing Merkel cells significantly decreased to 0.011% ± 0.007% already at P0 and even further to 0.006 ± 0.006% at P1. The decrease was significant between freshly isolated cells and P0 (*p* < 0.001), as well as between freshly isolated cells and P1 (*p* < 0.001). Bright field picture in Supplementary Figure S4 represents a typical morphology of human fetal keratinocytes at P0 and the positivity of Merkel cells for CK20 (green) and CK19 (red) (Supplementary Figures S3A–B’).

In addition to CK20, CK8 is also considered as a marker for Merkel cells. Therefore, we also investigated the co-expression of CK8 and CK20 in the freshly isolated epidermal cell mixture as well as in fetal keratinocytes at different passages (Figures 2A–C). We observed that in the freshly isolated epidermal cell mixture all CK20^+^ Merkel cells were in addition positive for CK8 (Figure 2A). Interestingly, CK8 expression in fetal epidermal cells increased markedly from isolation to higher (P0 and P1) passage numbers, but almost all CK8 positive cells were CK20 negative (Figures 2B,C). Therefore, we concluded that CK8 was not a suitable marker for detecting Merkel cells *in vitro* and therefore, we continued to use only CK20 as a marker for Merkel cells.

### Fetal skin derived Merkel cells co-express Piezo2 and neuroendocrine markers over several passages *in vitro*

**FIGURE 3:**
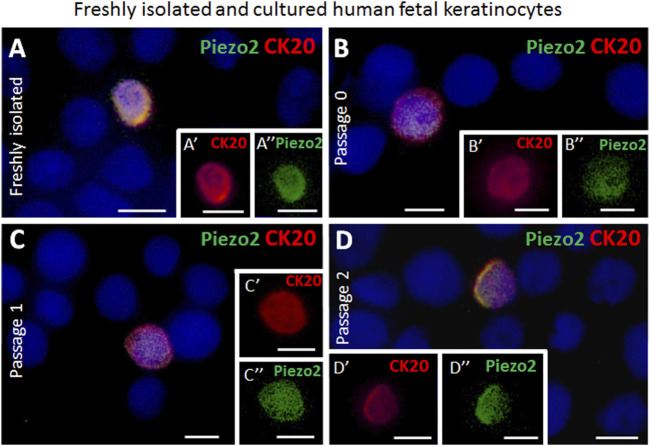
Examples of immunofluorescence staining for Piezo2 of freshly isolated and cultured cytospinned epidermal keratinocytes from human fetal skin. **(A,A′,A″)** Piezo2 (green) is expressed in a CK20-positive (red) Merkel cell in freshly isolated keratinocytes. **(A′)** depicts as magnification the CK20-positive (red) Merkel cell shown in **(A)**. **(A″)** highlights the Piezo2 (green) expression in the Merkel cell in **(A)**. **(B,B′,B″)** Piezo2 (green) is expressed in a CK20-positive (red) Merkel cell in culture (Passage 0). **B′** and **B″** show only the CK20 (red) or Piezo2 (green) expression of the Merkel cell displayed in **(B)**. **(C,C′,C″, D,D′,D″)** Piezo2 (green) is still expressed in CK20-positive (red) Merkel cells in cultured cells in higher passages (passage 1 in **C**, **C′**, **C″**, and passage 2 in **D**, **D′, D″**). **C′**, **D′**, **C″**, **D″** display single staining of Piezo2 (green) or CK20 (red) of the CK20-positive Merkel cell in **(C)** and **(D)**, respectively. Hoechst 33342 (blue) was used to counterstain nuclei. Scale bars: 10 μm.

**FIGURE 4:**
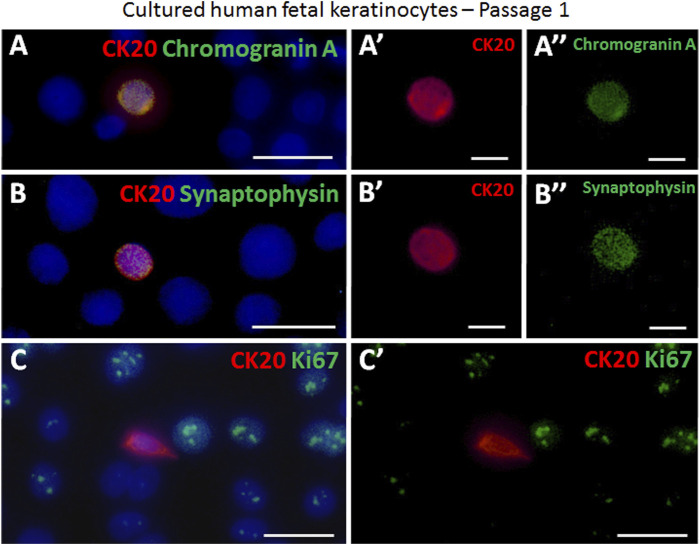
Examples of immunofluorescence staining of cultured cytospinned epidermal keratinocytes in passage 1. **(A,A′,A″)** CK20-positive (red) Merkel cell expressed chromogranin A (green). **(A′)** displays the CK20-positive (red) Merkel cell shown in **(A)**. **(A″)** demonstrates the chromogranin A (green) expression in the Merkel cell in **(A)**. **(B,B′,B″)** The neuroendocrine marker synapthophysin (green) is expressed in a CK20-positive (red) Merkel cell. **B′** and **B″** show as single staining the CK20 (red) or synaptophysin (green) expression of the Merkel cell displayed in **(B)**. **(C, C′)** Ki67 (green) and CK20 (red) expression in cultured cells. The proliferation marker Ki67 is not expressed in the Merkel cell. **(C′)** depicts Ki67 (green) and CK20 (red) double staining without cell nuclei Hoechst dye in **(C)**. Hoechst 33342 (blue) was used to counterstain nuclei. Scale bars: A, B, C, C’ = 25 μm; A′, A″, B′, B’’ = 10 μm.

The presence of the Piezo2 cation channel in Merkel cells has previously been demonstrated for human cells (García-Mesa et al., 2017; García-Piqueras et al., 2019). Here, we support this finding by revealing sustained co-expression of Piezo2 in CK20^+^ Merkel cells in freshly isolated epidermal cell mixture as well as over several passages (P0, P1 and P2) *in vitro* by immunofluorescence co-staining (Figures 3A–D). Moreover, neuroendocrine markers such as chromogranin A and synaptophysin (SYP) were continuously expressed in CK20 positive cells on cell culture plastic (Figures 4A–B’’). Taken together, these results show that Merkel cells retained their neuroendocrine character also *in vitro* after passaging.

Further, we analysed the expression of Ki67, a common proliferation marker indicating cells currently in the cell cycle (Sun and Kaufman, 2018). The majority of keratinocytes *in vitro* is actively dividing and hence positive for Ki67 (Figures 4C,C’). In contrast, we were not able to find any CK20^+^/Ki67^+^ double positive cell, implying that CK20 Merkel cells are not mitotically active during *in vitro* culturing.

### Fetal skin derived CK20 expressing Merkel cells are present in bioengineered skin substitutes *in vivo*

We then used cultured fetal epithelial cells and dermis derived fetal fibroblasts both at P1 to prepare dermo-epidermal skin substitutes. Further, we characterized the expression of Merkel cell markers 4 weeks after transplantation of these skin grafts onto immune-incompetent nude rats.

**FIGURE 5:**
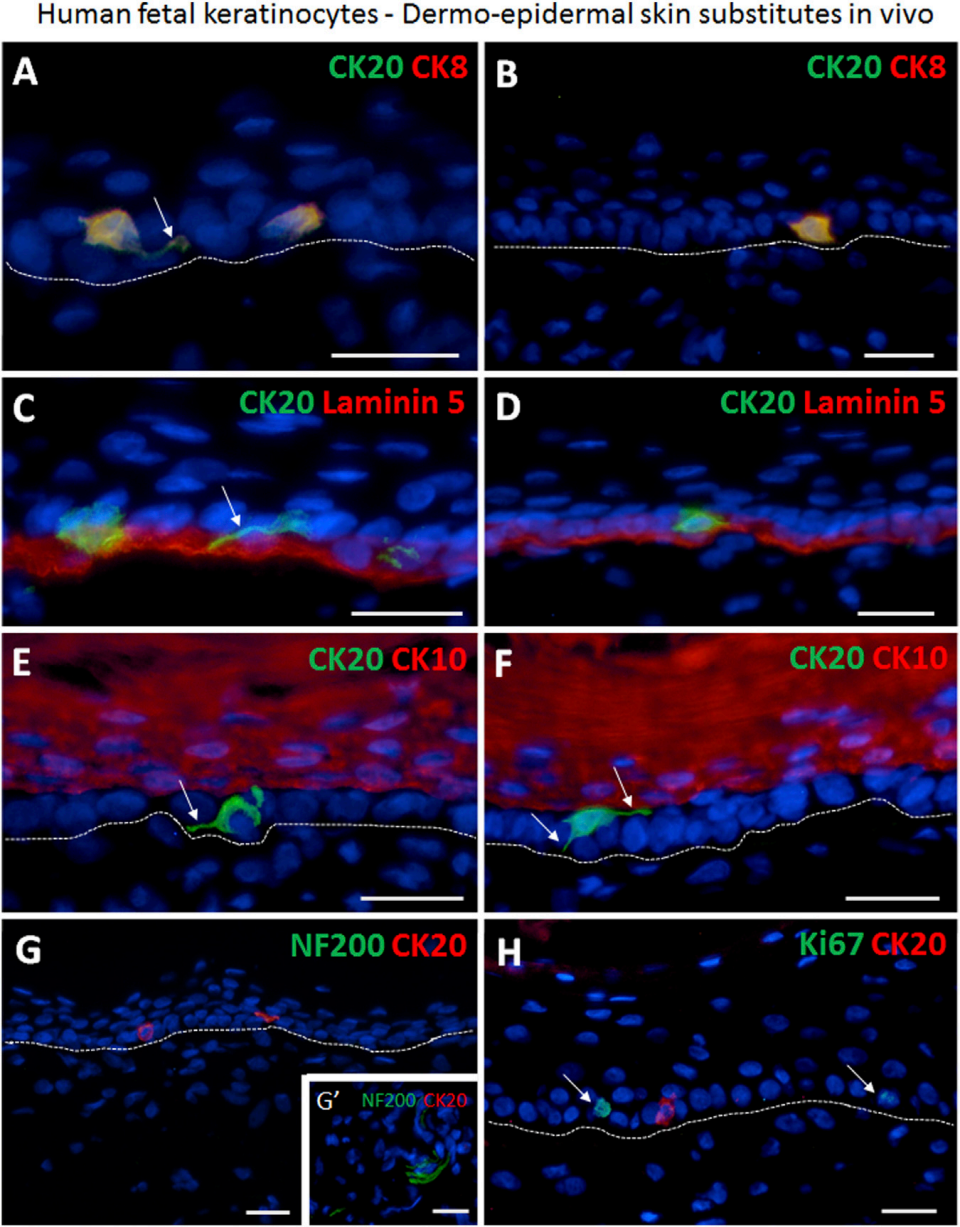
Immunofluorescence staining demonstrating the presence of human Merkel cells in human dermo-epidermal skin substitutes 4 weeks after transplantation in an animal model. **(A,B)** Examples of CK20 (green) and CK8 (red) double positive Merkel cells in the epidermis. Merkel cells are situated as single cells. Note that CK8 is expressed only in Merkel cells. White arrow depicts dendritic protrusion of a non-oval Merkel cell. **(C,D)** Basement membrane component Laminin 5 (red) and CK20 (green) expression. White arrow denotes dendritic protrusion of non-oval Merkel cell. **(E,F)** Suprabasal keratinocyte marker CK10 (red) and CK20 (green). CK20-positive Merkel cells reside in the basal layer of the epidermis. White arrows indicate dendritic protrusion of non-oval Merkel cells. **(G,G′)** Four weeks after transplantation, the CK20-positive (red) Merkel cells were still not associated with host nerves. **(G′)** shows a neurofilament 200 (NF200, green) positive host nerve still not projecting into the human dermis of the substitute serving as positive control for the staining. **(H)** Expression of Ki67 (green) and CK20 (red). The proliferation marker Ki67 is not present in the CK20-positive Merkel cell. White arrows mark Ki67-positive cells. Dashed white lines indicate locations of dermal-epidermal junctions. Hoechst 33342 (blue) was used to counterstain nuclei. Scale bars: 25 μm.

Most Merkel cells showed a migrating phenotype with dendritic protrusions in the transplanted human skin substitutes (Figures 5A, C, E–G). Strikingly, the prevalent expression of CK8 among the CK20-negative keratinocytes on culture plastic was not detected anymore *in vivo*. Therefore, CK8 could be considered as a marker for Merkel cells *in vivo* (Figures 5A,B). CK20/CK8 double positive cells were exclusively localized either in close proximity to or in contact with the basal lamina (Figures 5C,D). CK20 expressing cells were not observed in any of the stratified CK10-positive suprabasal layers (Figures 5E,F). Furthermore, Merkel cells mostly appeared as single cells, and only very rarely two or more cells were found adjacent to each other (Figures 5C–F). The quantification revealed that in tissue-engineered dermo-epidermal skin substitutes the number of CK20^+^ Merkel cells per 1 mm of basal cell layer is 1.88 ± 0.41 (Supplementary Figure S1B). This number is comparable to the number of Merkel cells in native human fetal skin (2.96 ± 1.27, *p* > 0.05, not significant).

In addition, even though early innervation from the host animal into the bioengineered dermo-epidermal skin were demonstrated based on NF200 immuno-staining, these nerve fibers were not yet associated with Merkel cells (Figure 5G,G’).

To confirm further the non-proliferative nature of Merkel cells *in vivo*, we investigated the expression of Ki67 in all CK20^+^ cells (Figure 5H). As observed in fresh isolations and *in vitro* cultures, CK20^+^ Merkel cells also did not express Ki67 in the skin substitutes *in vivo*.

### Fetal skin derived Merkel cells co-express Piezo2 and neuroendocrine markers in bioengineered skin *in vivo*

**FIGURE 6:**
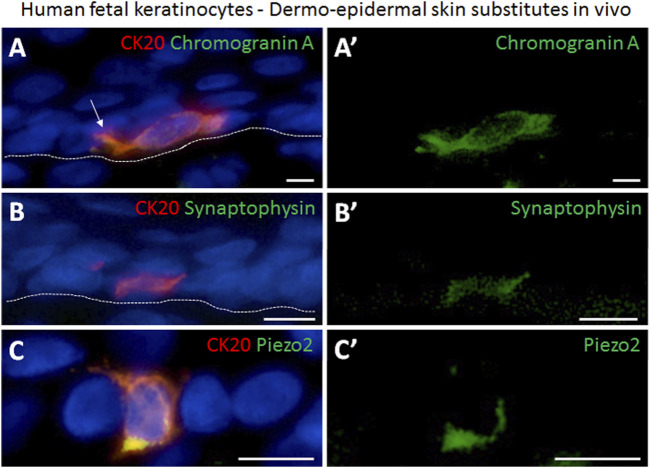
Immunofluorescence staining demonstrating the presence of neuroendocrine markers in human Merkel cells in dermo-epidermal skin substitutes 4 weeks after transplantation in an animal model. **(A,A′)** Expression of CK20 (red) and chromogranin A (green). **(A′)** single staining of chromogranin A (green) highlighting its presence in the CK20-positive Merkel cell in **(A)**. White arrow depicts dendritic protrusion of Merkel cell. **(B,B′)** CK20 (red) and synaptophysin (green) expression. **(B′)** staining of synaptophysin (green) demonstrating its presence in the CK20 positive Merkel cell in **(B)**. **(C,C′)** Piezo2 (green) expression in CK20-positive (red) Merkel cell. **(C′)** shows expression of Piezo2 in CK20-positive Merkel cell in **(C)**. Dashed white lines indicate locations of dermal-epidermal junctions. Hoechst 33342 (blue) was used to counterstain nuclei. Scale bars: 10 μm.

We further performed immunofluorescence staining to investigate the presence of mechanosensitive and neuroendocrine markers in Merkel cells in the human skin substitutes *in vivo*. We found that CK20-positive Merkel cells also displayed Chromogranin and synaptophysin, which are both considered as a marker for neuroendocrine cells (Figure 6A). In addition, Piezo2 was also identified in the CK20-expressing cells in the tissue-engineered skin substitutes confirming the identity of these cells (Figure 6C).

## Discussion

Merkel cells are considered as neuroendocrine cells responsible for the touch sensation in vertebrate skin. They are primarily localized in the basal cell layer of the epidermis, where they are in close contact with Aβ sensory neurons forming special Merkel cell-neurite complexes. Indeed, Merkel cells can be found in touch-sensitive areas of the skin, including lips, fingertips, as well as in touch domes of hairy skin (Oss-Ronen and Cohen, 2021).

Our study identified and characterized neuroendocrine Merkel cells in human fetal skin, in freshly isolated and cultured fetal epithelial cells and in fetal skin derived dermo-epidermal skin substitutes *in vivo*. The following aspects deserve to be addressed in more detail.

The presence and functionality of Merkel cells have been extensively studied in glabrous and hairy postnatal human skin (Fradette et al., 2003). However, only little is known about their exact distribution in the fetal skin. A previous study has shown that Merkel cells in the human fetal skin appear between 15 and 18 weeks of gestation (Boot et al., 1992). Moll et al. revealed in their study of fetal palmar skin that the Merkel cells were high in number in the human fetuses of 18–24 weeks of gestation and then their density gradually diminished in newborns and adults (Moll et al., 1984). In our study, we have reported about the location and distribution of Merkel cells in human fetal back skin at gestational weeks 24–25. We showed that directly after isolation, 0.037% ± 0.012% of all fetal epidermal cells were positive for the Merkel cell marker CK20 and that this number gradually decreased after sequential passaging *in vitro*. We have also shown, that human fetal Merkel cells express the recently described Piezo2 transmembrane protein (García-Mesa et al., 2017; García-Piqueras et al., 2019). Piezo2 has been reported as a mechanically activated cation channel playing a pivotal role in somatosensory mechanotransduction in Merkel cell-neurite complexes. Gracia-Mesa have recently revealed that 58.49% ± 2.2% of all fetal Merkel cells express Piezo2 at 22–23 weeks of gestation. Subsequently, this number gradually decreased and in 3 weeks old infant only 23.9% ± 1.1% of all Merkel cells were positive for Piezo2 (García-Mesa et al., 2022a). Interestingly, most of the Aβ type I slowly adapting mechanoreceptors that innervate Merkel cells were entirely negative for Piezo2. In addition, previous studies have revealed that mice deficient in Piezo2 in the skin exhibit reduced responses to gentle touch as well as increased sensitivity to pain (Ranade et al., 2014). Therefore, the presence of Piezo2 channels in fetal CK20-expressing Merkel cells and their close association with nerve endings suggest that these cells are able to mediate mechanotransductive signals leading to sense of touch in the skin of fetuses.

In our study, we were able to isolate and culture CK20 expressing Merkel cells from human fetal skin. We thereby observed that CK20 was restricted also *in vitro* only to Merkel cells that were also positive for neuroendocrine markers, such as chromogranin A and synapthophysin. Importantly, we detected the expression of the previously described Merkel cell marker CK8 in the majority of all keratinocytes *in vitro.* This demonstrates that CK8 is not exclusively expressed by Merkel cells *in vitro* (Moll et al., 1984; Moll and Moll, 1992). In this respect, the expression of another Merkel cell marker, namely CK18, was also observed previously in all keratinocytes in culture. Therefore, we proved that CK20 is the most reliable and unique marker for fetal Merkel cells. This result is in line with other studies that also reported CK20 as a reliable marker for non-fetal Merkel cells (Bose, 1994; Moll et al., 1995; Fradette et al., 2003; Tilling et al., 2014; Hahn et al., 2019).

Importantly, various studies convincingly proved that Merkel cells are of epithelial origin (Morrison et al., 2009; Van Keymeulen et al., 2009; Woo et al., 2010), despite the hypothesis that Merkel cells might be of neural crest origin (Grim et al., 2003; Szeder et al., 2003). The idea that epithelial cells give rise to Merkel cells originated from the observation that Merkel cells do not proliferate in human fetal skin (Moll et al., 1990; Moll et al., 1996). Accordingly, we showed that human fetal Merkel cells are not proliferative in culture and in skin dermo-epidermal substitutes after transplantation. Our observation corresponds to previous reports indicating that human non-fetal Merkel cells do also not proliferate *in vitro* and in skin substitutes after transplantation (Fradette et al., 2003; Hahn et al., 2019).

Although we convincingly showed that isolated and cultured Merkel cells keep their neuroendocrine characteristics *in vitro*, and that Merkel cells in dermo-epidermal skin substitutes continued to display these neuroendocrine properties, we could not detect Merkel cell-neurite complexes and eventually the mechanosensory function for fine touch sensation in dermo-epidermal skin substitutes *in vivo*. We investigated for the presence of heavy neurofilament 200 (NF200) in the skin substitutes, as the Aβ afferent nerves in Merkel cell-neurite complexes were reported to be constituted of heavy neurofilaments, not consisting of light or intermediate neurofilaments (Moll et al., 2005; Xiao et al., 2014). In this respect, previous observations showed that it takes at least 8–10 weeks to observe host nerve ingrowth into transplanted dermo-epidermal skin substitutes (Fradette et al., 2003; Biedermann et al., 2013; Biedermann et al., 2014; Biedermann et al., 2016; Hahn et al., 2019). In contrast, Hahn et al. (2019) postulated that the innervation of tissue-engineered skin is initiated within 4 weeks after transplantation, and that the association of Merkel cells with heavy neurofilament is observed only 6 weeks after grafting. Therefore, we speculate that the connection of Merkel cells and ingrowing nerve afferents requires more time in our skin substitutes after transplantation, and therefore could not be detected within the investigated time frame.

Importantly, we noted that the vast majority of Merkel cells in the dermo-epidermal skin substitutes displayed a non-oval dendritic shape. Our observation is emphasised by previous findings that non-oval dendritic Merkel cells are not in contact with nerve endings, and only Merkel cells in Merkel cell-neurite complexes display a typical oval non-dendritic shape (Nakafusa et al., 2006). Obviously, this finding supports the concept of Merkel cell heterogeneity. Some reports speculate that non-oval dendritic Merkel cells are a population of non-matured Merkel cells still striving for a connection to nerve endings, whereas oval non-dendritic cells display matured Merkel cell population, which is in close contact with afferents (Tachibana et al., 1997; Nakafusa et al., 2006).

Besides the lack of connection between Merkel cells and afferent nerve endings, out study represents some other limitations. Importantly, we have analysed fetal human skin samples at 23–25 gestational age for the presence and density of Merkel cells. Therefore, we could not precisely estimate the time point at which Merkel cells appear in skin during fetal development, and how the number of Merkel cells changes during embryogenesis. In addition, our study lacks a functional assays that could confirm the restoration of fine touch sensation in tissue-engineered skin after transplantation.

In conclusion, our study clearly demonstrates that it is possible to isolate and culture human fetal Merkel cells and that these cells can be detected in fetal dermo-epidermal skin substitutes after transplantation. These findings are important for the preparation of dermo-epidermal skin substitutes using fetal-skin derived fibroblasts and keratinocytes. This findings are of pivotal importance for the future application of such tissue-engineered skin in clinics.

## Data Availability

The raw data supporting the conclusion of this article will be made available by the authors, without undue reservation.
