# Supplementary material for: Human fetal skin derived merkel cells display distinctive characteristics in vitro and in bio-engineered skin substitutes in vivo
Source: Front Bioeng Biotechnol. 2022 Sep 15;10:983870. doi: 10.3389/fbioe.2022.983870 (PMC9520781; doi:10.3389/fbioe.2022.983870)
Supplement: Supplementary file 1 [file DataSheet1.pdf]

Supplementary Material

1. Supplementary Figures

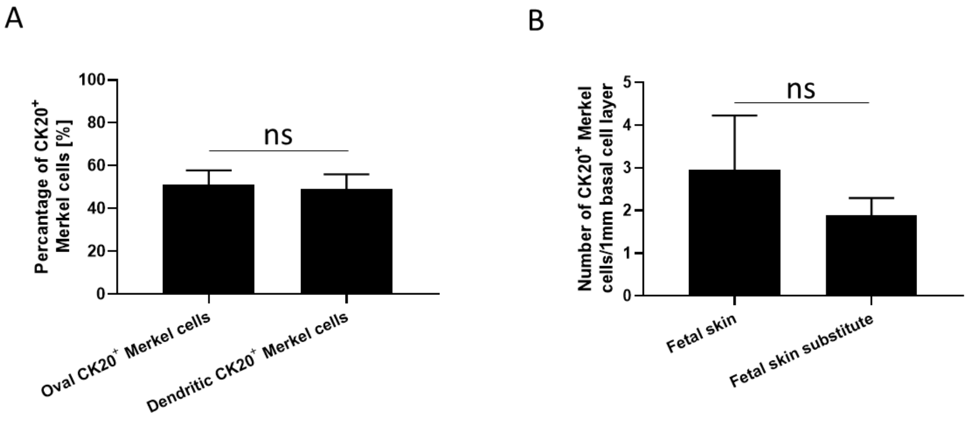

Supplementary Figure 1. Quantification of Merkel cells. (A) Quantification of oval and dendritic Merkel cells in human fetal skin. Note that 50.9±6.8% of all $CK20^+$ Merkel cells have an oval phenotype, while 49.1±6.8% of all $CK20^+$ Merkel cells exhibit a dendritic phenotype. $p>0.05$, not significant. P value calculated using unpaired student t-test. **(B)** Quantification of $CK20^+$ Merkel cells in human fetal skin as well as in tissue-engineered skin constructed with fetal skin derived keratinocytes and fibroblasts. Note that fetal skin contains 2.96±1.27 of $CK20^+$ Merkel cells per 1mm of basal cell layer, while tissue-engineered skin consists of 1.88±0.41 of Merkel cells per 1mm of basal cell layer. $p>0.05$, not significant. P value calculated using unpaired student t-test.

Human fetal skin - «dendritic» Merkel cell without nerve ending contact

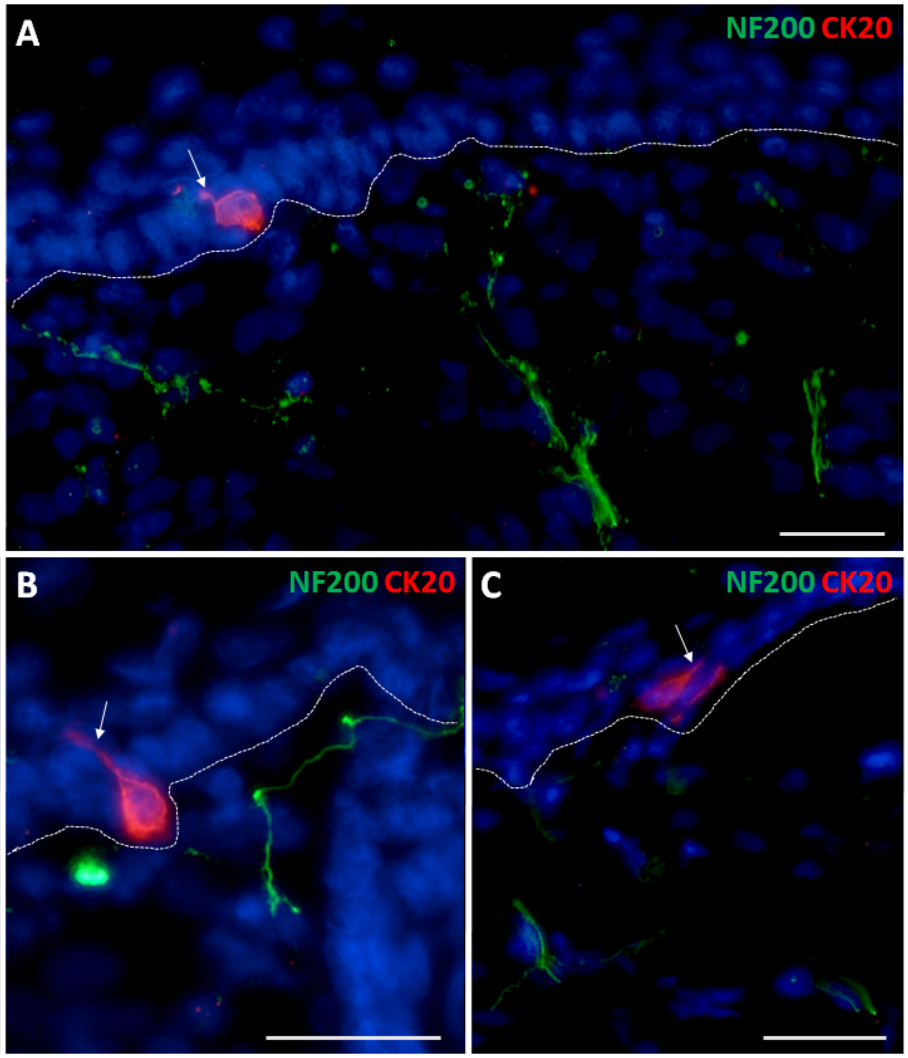

Supplemental Figure 2. Examples of immunofluorescence staining of dendritic Merkel cells not associated with nerves in human fetal back skin. (A, B, C) Dendritic non-oval CK20-positive (red) Merkel cells are not in contact with nerve-ending projections from neurofilament 200 (NF200, green) positive nerves. White arrows depict dendritic protrusion of non-oval Merkel cells. Dashed white lines indicate locations of dermal-epidermal junctions. Hoechst 33342 (blue) was used to counterstain nuclei. Scale bars: 25 µm.

Human fetal skin - Non «dendritic» Merkel cell with nerve ending contact

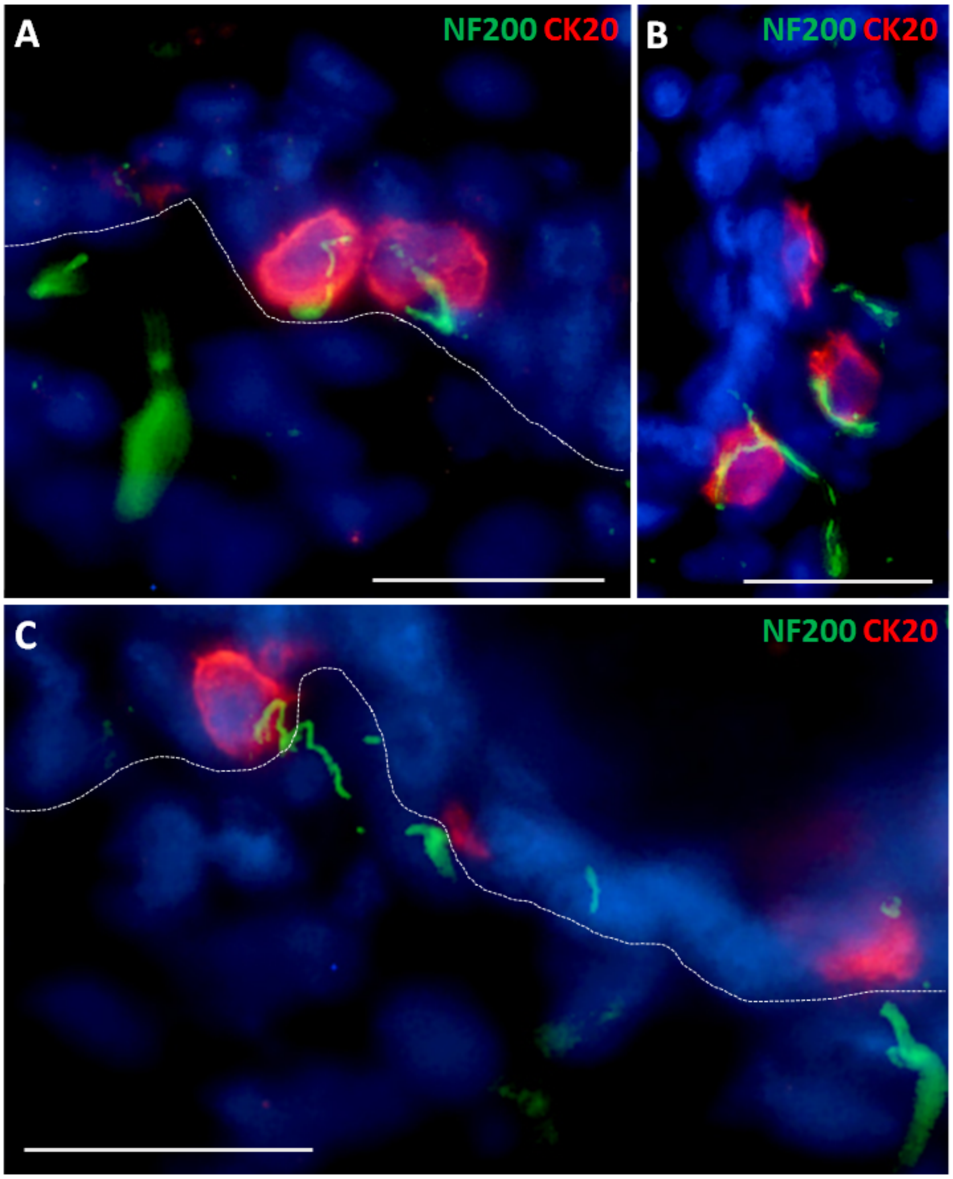

Supplemental Figure 3. Examples of immunofluorescence of non-dendritic oval Merkel cells associated with nerves in human fetal back skin. (A, B, C) Non-dendritic oval CK20-positive (red) Merkel cells are in close contact with nerve-ending projections from neurofilament 200 (NF200, green) positive nerves. Dashed white lines indicate locations of dermal-epidermal junctions. Hoechst 33342 (blue) was used to counterstain nuclei. Scale bars: 25 µm.

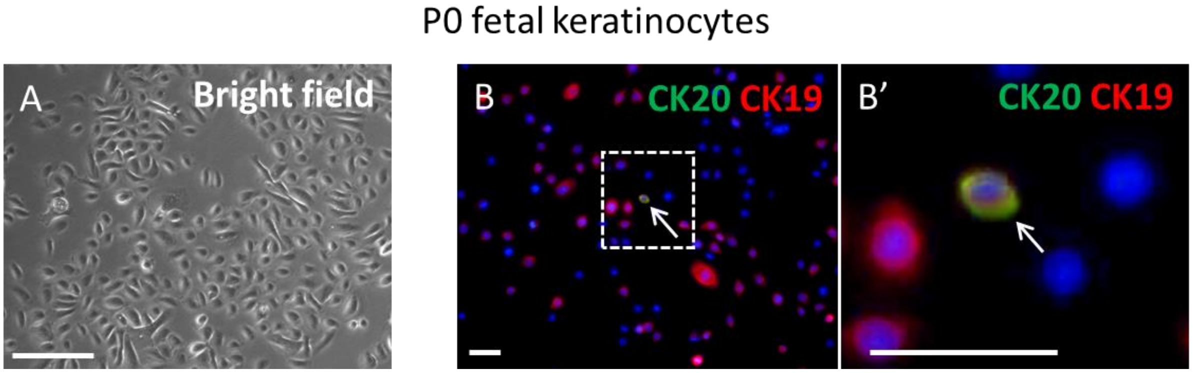

Supplementary Figure 4. Morphology of fetal cultured keratinocytes. (A) Microscopic bright field picture showing the morphology of human fetal keratinocytes at passage 0 (P0). Scale bar: 100 μm. **(B-B')** Immunofluorescence pictures showing the expression of CK20 (green) and CK19 (red) in human fetal keratinocytes at P0. Please note the presence of CK20 and CK19 positive Merkel cells. Picture in B' represents the higher magnification of picture in B. Scale bars: 50 μm.
